# Pathology of Gout, Apoplexy, &c. Founded on the Blood's Vitality

**Published:** 1825-05

**Authors:** John Jones


### Art. VII.—*Pathology of Gout, Apoplexy, &c. founded on the Blood's Vitality.* By John Jones, Esq.

Gout.—Venous congestion of the abdominal viscera, *gradually* and *progressively* formed, constitutes the proximate cause of this disease, which usually occurs at that period of life when venous plethora has commenced, and consequently when the vigour of the constitution is beginning to decline. This predisposition, sooner or later, occurs in every one; but is greater, and exists earlier, in some than others, from causes depending on original formation, peculiar habits of the patient, &c. Thus it is greatly increased by habitual intemperance, either in eating or drinking, particularly when conjoined with sedentary pursuits; and we find, therefore, the studious man and bon-vivant equally obnoxious to gout. The veins being largely supplied with blood, by ingestion of superabundant nutriment, the action of the heart and arteries being at the same time weakened by want of exercise in one, and indirect debility, induced by habits of intemperance, in the other,—venous congestion of the abdominal viscera becomes established, accompanied sooner or later by a paroxysm, which appears nothing more than *reaction* caused by the gradual increase of visceral congestions, in conjunction with venous plethora. The extremities are the most frequent seat of attack, from the circulation being weaker in these parts, and consequently more subject to irregularities. When organic congestions are *suddenly* formed, as is the case in fever, the consequent depression of the vital energies is also sudden and proportionably great, and the whole vascular system becomes morbidly excited, constituting febrile reaction. When congestions are *gradually* formed, the depression is also gradual, and the subsequent reaction is for the most part inflammatory, constituting a paroxysm of gout.

In considering reaction an effect of diminished nervous energy, which causes an accumulation of materia vitæ diffusa in the arterial blood, as explained in treating of the pathology of Fever, it appears a process pursued by nature for relieving the vascular system from a morbid accumulation of vitality, which becomes expended by the increased arterial action, constituting reaction. We may thus account for the relief afforded to the constitution by many eruptions, particularly those which appear critical, and precede convalescence from acute diseases. In addition to the above cause, febrile or gouty reactions might afford relief to the system by the increased action of the heart and arteries which accompanies them, accelerating the progress of blood in the veins, and thus tending directly to remove the congestions on which the disease depends. The spontaneous subsidence of fever in many instances, and likewise that of a gouty paroxysm, might be thus explained, which has been observed by Sydenham to be short in proportion to its violence. On similar principles, also, we may account for the improvement to the general health, which is supposed to follow a fit of the gout. The late Dr. Parry, in his Elements of Pathology, observes, " Examples have occurred in which a patient, accustomed to vernal gout, and missing the usual fit, has had erysipelas, the next spring hæmorrhoids, and the following spring a fever, cured by blood-letting, each with equal relief to the constitution.

Gout is generally considered a constitutional disease, and, when once occurring, as apt to return at intervals for the remainder of life. This disposition to return might be explained in the following manner:—When venous congestions have been gradually formed, as is the case in gout, the venous parietes become weakened in consequence of protracted distention, and therefore, although they may be relieved from existing congestions, a disposition to be similarly affected continues, and the disease inevitably returns, if the exciting causes are not avoided: and we find, therefore, that the prophylactic treatment most useful is such as tends to obviate the disposition to congestion in the chylopoietic viscera.

Dr. Scudamore, who has written so ably on Gout, considers it as depending on congestion of the abdominal viscera; in which opinion he is supported by the physiological fact, that venous plethora and venous congestions necessarily produce debility, accompanied for the most part, sooner or later, by reaction. Instead, therefore, of being a disease of the nervous system, as asserted by Cullen and taught by the schools, it appears more correct to consider gout as originating in the vascular system, and the nerves as only secondarily affected.

The different forms of gout, like those of idiopathic fever, seem to depend on the relative state of the vital powers, in connexion with visceral congestions. They are—

1. *Regular gout;* in which the inflammatory reaction on the extremities is well developed.

2. *Atonic;* in which the vital energies are too feeble to support vascular action, when the disease produces great derangement in the functions of the chylopoietic viscera.

3. *Retrocedent;* in which inflammation, having occurred on the extremities, suddenly recedes, and some internal organ, perhaps of vital importance, is deprived of its energies, accompanied with symptoms of the atonic state.

4. *Misplaced;* in which the gouty reaction does not occur on the extremities, but affects some internal organ with inflammation.

Visceral congestions have been shown to constitute the proximate cause of gout as well as fever, and followed by either disease, according to their sudden or slow production. It might be observed, also, that in fever the brain is generally more or less affected, causing a greater derangement in the functions of the nervous system than occurs in gout, in which congestions are confined, for the most part, to the abdominal viscera.

Defective reaction indicates the degree of danger in both diseases, and is the chief cause of their modifications. When deficient in fever, the worst forms of that disease are the consequence: when defective in gout, the disease assumes the atonic form, in which the system becomes greatly deranged; or it is retrocedent, affecting, perhaps, some internal organ of vital importance, which is suddenly deprived of its energies, in consequence of its veins being gorged with blood, by which it is rendered incapable of performing its functions. When this state occurs in the heart, frightful syncopes are the consequence; when in the lungs, asthma is produced; and, if the brain is thus affected, apoplexy follows, which, if not relieved, speedily proves fatal. The stomach is generally more or less affected in every form of gout, but, when retrocedent or atonic, it is subject to violent spasmodic attacks, in which the pain is exceedingly acute, accompanied with great and general depression. This dangerous affection of the stomach is doubtless the effect of diminished nervous power in that organ, produced in connexion with existing congestions.

*Treatment.*—The phenomena of gout appear satisfactorily explained by the above view of its pathology. In considering it a constitutional disease, depending on venous congestion of the abdominal viscera, gradually induced, by which the venous parietes are morbidly distended, it is evident that, the longer the disease has continued, the paroxysms are likely to be more frequent, and a permanent cure less probable. We accordingly find that, when it has been of long standing, the constitution impaired by reiterated paroxysms, debauched habits, or old age, little can be done for the relief of the afflicted patient, but to palliate urgent symptoms. When, on the contrary, the patient is young, has a vigorous constitution, and has had but few paroxysms, the progress of the disease might be checked, and a recurrence of the paroxysms be wholly prevented, by a rigid observance of prophylactic measures, which consist in a well-regulated diet and active exercise. That diet which most disposes to plethora, may be considered most favourable to the production of gout; and, as animal food and fermented liquors have a peculiar tendency to produce plethora, we find that abstaining from them is the surest means of preventing a return of the paroxysms. I recollect the late Dr. Gregory observing in his Lectures, that he was a striking instance of the beneficial effects produced by abstaining from animal food. The predisposition to gout was so great, that, when seventeen years old, he had a severe attack; but, by persevering abstinence, he had wholly prevented a relapse. Fermented liquors and tartarous wines are supposed peculiarly to favour the production of gout, independently of inducing plethora. It appears, however, very questionable whether this opinion is founded on fact, as it is well known that malt liquor is the principal beverage of the labouring classes in most of our inland counties, and yet amongst them it is a rare disease. On the contrary, it is by no means unfrequent with those who even abstain from fermented liquors, but are of sedentary habits, and indulge in the pleasures of the table.

To insure the good effects of a well-regulated diet, exercise is indispensable. Haller observes, "The veins are placed near the muscles, which by their swelling compress the interposed vessels, and since every pressure of the veins determines the blood towards the heart, therefore all this force is entirely employed in accelerating the blood to the heart." This explains the great utility of exercise in preserving the vigour of the constitution, and proves it to be an established law of nature, that moderate labour is essential to the preservation of health; for, although the energies of life may be impaired by excessive labour, and premature old age, with all its infirmities, be the consequence, yet the decree of the Deity, that man should gain his bread "by the sweat of his brow," may be considered as a blessing rather than a curse, in our present state of existence. Indolence impedes the organic functions, undermines the fountains of health, and gradually, but inevitably, leads to disease. We accordingly find that those persons who are obliged to labour for their living are generally strangers to gout, which is justly considered the offspring of indolence and free living.

Medicines are only applicable to the relief of the paroxysms, and can have little or no effect in preventing their recurrence. The indications are—1, to lessen or remove congestions of the abdominal viscera; 2, to allay irritation produced by the inflammatory reaction; 3, to restore the tone of the stomach, the functions of which are generally more or less deranged. The remedies usually employed sufficiently fulfil the above indications, and consist in purgatives, diaphoretics, diuretics, sedatives, and stomachics; some of which it may be sufficient at present to consider, in connexion with the preceding views.

*Purgatives.*—This class of remedies has a direct tendency to relieve congestions of the abdominal viscera, and at the same time, by equalising the circulation, has a powerful effect in allaying irritation. But, should they fail in diminishing existing congestions, symptoms of the atonic state will probably be the consequence; and, therefore, active purgation, if not decidedly beneficial, might be highly injurious, by unnecessarily reducing the energies of the system. Thus we may account for the great estimation in which certain nostrums have been held for a time, and afterwards discarded as extremely pernicious. The famous medicine Eau Medicinale was of this description; and in many instances, no doubt, it merited the praise bestowed upon it, particularly when administered on first attacks of the disease, or before the constitution was impaired by age and frequent returns of paroxysms. Its indiscriminate employment, however, subjected it to the opprobrium of a poison, and we now agree with the French in considering it a medicine too dangerous to be admitted into general practice. It is accordingly superseded by the colchicum autumnale, as the fashionable remedy of the day; the effects of which are similar, but less violent, to those of the eau medicinale, of which it is supposed to have been the active constituent. The employment of purgatives, although requiring considerable caution, are indispensable in the treatment of gout. The bowels require to be constantly, but moderately, acted upon by warm laxatives, occasionally conjoined with calomel and antimony. In the employment of purgatives, as in most other cases, we find the truth of the maxim, "Medio tutissimus ibis."

*Sedative applications.*—To allay the extreme pain attending gouty inflammation appears very desirable. If the disease were merely local, as is the opinion of some physicians, we might, without hesitation, employ all the resources of medicine for subduing the inflammatory action. But, in considering it a constitutional disease, and the local inflammation induced sympathetically with internal congestions, we find, both by theory and practice, that such means cannot be employed without considerable hazard. If the inflammatory action is great, and the patient young and plethoric, the antiphlogistic regimen, rigidly pursued, is usually necessary; and in such cases even the application of cold water, as recommended by Dr. Kinglake, might afford relief, without being followed by bad consequences. But if the disease has been of long standing, the patient advanced in years, and the local inflammation not great, indicating imperfect reaction, the application of repellants is doubtless extremely hazardous, lest it become retrocedent, and affect some internal organ of vital importance. If repellants are ever safe, they are only so when there is reason to suppose that organic congestions have been subdued by previous treatment; and then they are seldom necessary, as the inflammation spontaneously subsides when the cause on which it depends is removed.

Apoplexy.—The symptoms characterising this disease are indicative of almost a total impediment to the development of nervous energy. It might be the effect of—1, mechanical pressure on the brain, preventing the due performance of its functions; 2, congestion of the cerebral veins, by which the brain is prevented deriving vitality from the blood, and communicating it to the system; 3, narcotic poisons, which act directly on the nervous system, depriving it of its energies, and consequently rendering it incapable of supporting the process of life.

1. *Mechanical pressure.*—The most frequent causes are fracture of the skull, by which a portion of bone is forced upon the brain; effusion of blood, by rupture of some of the cerebral vessels, or of water proceeding from the exhalents, in consequence of vascular distention. By whatever means pressure on the brain is produced, the symptoms are similar, constituting either complete apoplexy, in which all the voluntary powers of life are suspended, or paralysis, when only part of the body is deprived of its energies, which is usually on the opposite side to that on which the cerebral pressure exists. When apoplexy is the consequence of effusion, it generally proves fatal; or, if the patient recover from the fit, a state of paralysis exists, perhaps, for the remainder of life. It frequently, however, happens that patients are affected with an apoplectic fit, which, by appropriate means, soon subsides, and recovery is as complete as if it had never occurred. Such cases are supposed to depend on mechanical pressure upon the brain, produced by the distended vessels. But it appears to me doubtful whether compression from such a cause could be so great as *alone* to produce a fit of apoplexy.

2. *Congestion of the cerebral veins.*—In addition to the effects of pressure from this state of the cerebrum, the circulation in the brain being nearly suspended, the blood is prevented from undergoing the changes necessary for the continuance of vitality, and the supplies of nervous energy to the system is consequently obstructed. Apoplexy produced by these combined causes subsides on removing the vascular distention of the brain, the functions of which being no longer obstructed, the operations of life are continued as before.

As the energies of the system are nearly obliterated in this disease, reaction seldom occurs, or only to a partial extent; and therefore fever rarely accompanies apoplexy, or, when it does, it is, as might be expected, generally of the typhoid type. A lady whom I attended had been long afflicted with paralysis of the lower extremities, but in other respects enjoyed tolerable health. She was suddenly attacked with all the symptoms of sanguineous apoplexy, together with those of the most malignant typhus: tongue nearly black, and perfectly dry, with dark sordes on the teeth, &c. In this state she continued for a few days, when the remaining spark of life became extinguished.

3. *Narcotic poisons* may be classed under two heads—1, vegetable; 2, gaseous.

1. *Vegetable narcotic poisons.*—These act either directly on the nervous system, and render it incapable of deriving vitality from the blood, or on the blood itself, through the medium of the absorbents; by which cerebral congestions are induced, causing symptoms of apoplexy. The almost instantaneous effects of hydrocyanic acid appear produced by its direct action on the nervous system. Opium, from its slow operation, and causing the usual symptoms of apoplexy, seems to be received into the circulation, and to produce its peculiar effects by impeding the progress of blood in the cerebral vessels. The poisonous effects of opium, and the symptoms of apoplexy, are so similar, that there is little doubt but the same state of the brain exists in both; and we find, in post-mortem examinations, in each the same distention of the cerebral vessels.

2. *Gaseous narcotic poisons.*—All air deprived of oxygen are incapable of supporting animal life, and consequently poisonous; and the most noxious of the gases, if well diluted with atmospherical air, are comparatively harmless. As oxygen is the source of vitality, life must, of course, cease by its absence. It is therefore questionable whether gases, reputed noxious, possess in themselves any peculiar property which renders them so; or if they should, asphyxia might doubtless be the effect of the mere absence of oxygen in the respired air, without the aid of a specific poison. In such cases, death must necessarily be as certain and as sudden as if occasioned by strangulation.

CONCUSSION.—The symptoms produced by this state of the brain are similar to those of apoplexy, but in a less degree, of which it may be considered a modification. The functions of the brain are so much impeded by the shock which has caused the concussion, that the action of the heart and arteries is considerably weakened, which, together with the injury the brain has sustained, disposes to venous congestion of that important organ, and the vital energies become almost extinct. But, if there are no organic lesions, the sensorial functions are gradually restored, and if reaction occurs, the consequent fever is of the typhoid type; although fever is not a necessary consequence of concussion, as its peculiar symptoms not unfrequently wholly subside when the cerebral derangement is removed.

VARICOSE VEINS.—In this disease venous congestion is evident to the senses, and debility, as might be expected, is the consequence. But, as the cause is of a local nature, the effects are also limited in their extent. The weakness is confined to the limb so affected, the temperature of which is considerably diminished; and, if affected with inflammation, it is erysipelatous. Indolent ulcers often accompany the disease, which, together with the morbid state of the veins, are exceedingly difficult of cure. The operation recommended by Mr. Brodie, in the 7th volume of the Medico-Chirurgical Transactions appears entitled to more consideration than it has hitherto obtained: it consists in passing a narrow bistoury, with its flat surface, between the integuments and one of the largest of the diseased veins, which is opened by slightly turning the sharp edge of the instrument, so as to bring it in contact with the vein. The hemorrhage is easily controlled, and, by the application of a well-adapted bandage, a permanent cure is obtained. The vigour of the limb is soon restored; the ulcerations assume a healthy aspect; and this troublesome disease is speedily removed, after having tormented the patient, perhaps, for years.

The efficacy of this operation is also strikingly illustrated by some cases published in the Edinburgh Medical and Surgical Journal for January 1820, communicated by my friend, John Foster, now Beard, esq. one of the surgeons to the Infirmary at Newcastle-upon-Tyne.

[To be continued.]

